# Bandwidth and Gain Enhancement of a CPW Antenna Using Frequency Selective Surface for UWB Applications

**DOI:** 10.3390/mi14030591

**Published:** 2023-02-28

**Authors:** Musa Hussain, Md. Abu Sufian, Mohammed S. Alzaidi, Syeda Iffat Naqvi, Niamat Hussain, Dalia H. Elkamchouchi, Mohamed Fathy Abo Sree, Sara Yehia Abdel Fatah

**Affiliations:** 1 Department of Electrical Engineering, Bahria University Islamabad Campus, Islamabad 44000, Pakistan; 2 Department of Information and Communication Engineering, Chungbuk National University, Cheongju 28644, Republic of Korea; 3 Department of Electrical Engineering, College of Engineering, Taif University, P.O. Box 11099, Taif 21944, Saudi Arabia; 4 Telecommunication Engineering Department, University of Engineering Technology, Taxila 47050, Pakistan; 5 Department of Smart Device Engineering, Sejong University, Seoul 05006, Republic of Korea; 6 Department of Information Technology, College of Computer and Information Sciences, Princess Nourah bint Abdulrahman University, P.O. Box 84428, Riyadh 11671, Saudi Arabia; 7 Department of Electronics and Communications Engineering, Arab Academy for Science, Technology and Maritime Transport, Cairo 11865, Egypt; 8 Department of Electronics and Communication, Higher Institute of Engineering and Technology, EI-Tagammoe EI-Khames, Cairo 11835, Egypt; 9 Department of Electrical Engineering, Faculty of Engineering, Egyptian Chinese University, Cairo 11771, Egypt

**Keywords:** UWB antenna, compact antenna, FSS reflector, gain improvement, high gain antenna

## Abstract

In this article, a single-layer frequency selective surface (FSS)-loaded compact coplanar waveguide (CPW)-fed antenna is proposed for very high-gain and ultra-wideband applications. At the initial stage, a geometrically simple ultra-wideband (UWB) antenna is designed which contains CPW feed lines and a multi-stub-loaded hexagonal patch. The various stubs are inserted to improve the bandwidth of the radiator. The antenna operates at 5–17 GHz and offers 6.5 dBi peak gain. Subsequently, the proposed FSS structure is designed and loaded beneath the proposed UWB antenna to improve bandwidth and enhance gain. The antenna loaded with FSS operates at an ultra-wideband of 3–18 GHz and offers a peak gain of 10.5 dBi. The FSS layer contains 5 × 5 unit cells with a total dimension of 50 mm × 50 mm. The gap between the FSS layer and UWB antenna is 9 mm, which is fixed to obtain maximum gain. The proposed UWB antenna and its results are compared with the fabricated prototype to verify the results. Moreover, the performance parameters such as bandwidth, gain, operational frequency, and the number of FSS layers used in the proposed antenna are compared with existing literature to show the significance of the proposed work. Overall, the proposed antenna is easy to fabricate and has a low profile and simple geometry with a compact size while offering a very wide bandwidth and high gain. Due to all of its performance properties, the proposed antenna system is a strong candidate for upcoming wideband and high-gain applications.

## 1. Introduction

With the rapid advancements in wireless communication technology, the current and impending communication systems necessitate electrically small, geometrically simple, and low-profile antennas with high gain and wideband characteristics [1,2]. Due to promising radiation characteristics such as higher data rate, large bandwidth, and minimal power requirement, ultra-wideband (UWB) antennas are considered as auspicious candidates for various commercial and military applications such as health monitoring systems, radar imaging, tracking, and precision locating applications [3,4,5,6,7,8]. However, some of these applications require high-gain antennas with increased directivity [9,10]. In recent years, various works involving different methodologies such as stub loading, slotting, electromagnetic band gap structures (EBGs), and metasurfaces have been reported in the literature to enhance the gain of the UWB antennas [11,12,13]. Furthermore, the gain of the antenna can also be enhanced by manipulating the near-fields or by using novel materials including graphene, but this will result in a high-cost system [14,15].

In addition to the aforementioned techniques, frequency selective surfaces (FSSs) have been investigated recently for gain improvement. FSSs based on artificial intelligence are used for gain enhancement, however, these types of FSS require a lot of knowledge and coding skill which are time-consuming [16]. Thus, a numerical analysis-based FSS has been used for reflection, transmission, or absorption of EM waves and is used as a band-pass or absorber, respectively [17,18]. Several works have reported FSS-loaded UWB antennas for 5G and future 6G wireless communication devices [19,20,21,22,23,24,25,26,27,28,29,30,31,32,33,34,35]. In [19], a geometrically simple FSS-loaded antenna is reported for high-gain and UWB applications. Although the design has a compact dimension of 26 mm × 26 mm, the overall size is enhanced after loading the FSS layer. Another simple and compact antenna design employing FSS is reported in [20]. The reported work has a narrow bandwidth of 0.3 GHz ranging from 3.6 to 3.9 GHz, and no significant improvement in gain is observed. Another microstrip patch antenna with a compact overall size of 45.8 mm × 55 mm × 10 mm after FSS loading and operating over UWB of 2.9–9.3 GHz is presented in [21]. The reported design is compact and has a wide operating band, however, only 2 dBi (3.12 dBi to 5.12 dBi) improvement in gain is observed. Another UWB antenna operating on a 4.7–14.9 GHz band and loaded with a single-layer FSS structure to enhance the gain is proposed in [22]. This proposed geometry has overall large dimensions. In [23], an altered circular loop-shaped FSS-loaded antenna is reported for 5G applications. A significant improvement in gain by 4 dB is obtained by incorporating FSS, however, the overall size of the proposed structure is large (98 mm × 98 mm × 31.8 mm), and the operational band is also comparatively narrow, ranging from 3.6 to 6.1 GHz. In another work [24], a high-gain and wideband antenna operating at the millimeter-wave band is presented. This antenna has the advantages of high gain but has the demerits of complex geometry and large overall size. Another wideband FSS-loaded antenna in [25] has an operational bandwidth of 3–12 GHz and a peak gain of 6.8 dBi, but the dimensions of the proposed structure are large, reducing the suitability of this antenna for future smart devices. Similarly, a few other works [26,27,28,29,30] reported compact and geometrically simple antennas for UWB frequencies, incorporating single-layered FSS for gain improvement. These antennas are proposed for various applications such as WiMAX, 5G sub-6 GHz, C-band, S-band, and X-band applications. These reported designs either have large dimensions or do not show significant gain improvements. Moreover, in [31], an antenna with a double-layered FSS operating over a 3.14–4.64 GHz band is reported with a gain enhancement of 8.7 dBi. Although the antenna attained a wide operational band and high peak gain, the design complexity of the proposed structure is increased as double layers of FSS are employed. Another double-layered FSS-loaded antenna with an operational band ranging from 3–13.4 GHz is reported in [32]. The incorporation of FSS improved antenna gain with a peak value at 8.5 dBi over the operational frequencies. This design also has more design complexity due to double FSS layers.

On the other hand, various single-layered FSS-loaded antennas are reported in the literature [33,34,35,36,37,38]. The UWB antenna proposed in [34] employed a single-layered FSS to improve gain. The proposed antenna attained a gain improvement of 2–3.5 dB with peak gain of 7.6 dB for the resonant band. In [35], another UWB antenna loaded with a single layer of FSS is reported. A gain improvement of 2.5–5.2 dBi is achieved for this configuration. Another work [36] presented a monopole UWB antenna for radar and imaging applications, with FSS to improve gain. Likewise, in [38], the reported antenna is a UWB antenna with tightly coupled FSS. This work uses the squirrel search algorithm (SSA) to optimize the design parameters. It is observed that the works discussed above either have complex geometries due to dual-layered FSS or have low gain enhancement. These multi-layered structures have limited applications due to the increased size and design complexity.

Considering the limitations and discrepancies observed in previously reported works, this work proposes a simply shaped, compact, ultra-wideband, low-profile, and high-gain FSS-loaded patch antenna for WiMAX, 5G sub-6 GHz, C-band, S-band, and X-band applications used for 5G and future 6G communicating devices. The rest of the article is split into three sections. In Section 2, the design methodology of the presented UWB antenna and unit cell of the FSS is discussed along with a parametric analysis of key parameters. In Section 3, the measured and the software-predicted results of the antenna are compared with and without the FSS structure. The comparison of the suggested design with the earlier reported design is listed in Table 1, to express the potential of the proposed FSS-loaded UWB antenna. The work is concluded in the fourth section, along with references.

## 2. Design and Methodology of Proposed FSS-Loaded Ultra-Wideband Antenna

In this section, the design of the proposed ultra-wideband antenna as well as proposed FSS, along with design stages and optimization algorithm, is discussed. The performance of the antenna, as well as parametric analysis of key parameters, is also explained in this section.

### 2.1. Design of Ultra-Wideband Antenna

**Figure 1:**
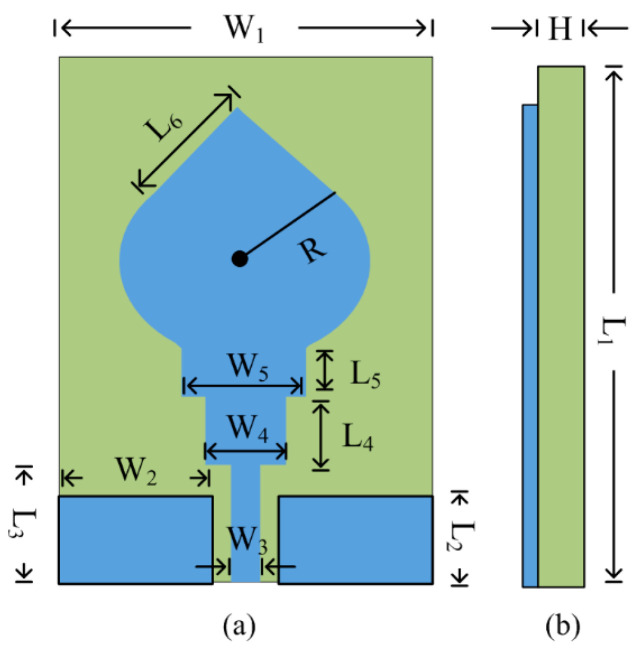
Structure of suggested ultra-wideband antenna design: (**a**) front view (**b**) side view.

Figure 1 shows the structure of the suggested ultra-wideband antenna suitable for numerous high-gain and wideband wireless devices. The proposed antenna contains a coplanar waveguide (CPW) feedline and the multi-stub-loaded hexagonal patch. The stubs are added to the primary antenna in order to obtain ultra-wideband and high gain. The CPW feeding technique is adopted with the advantages of low dispersion and uniplanar configuration. The impedance matching of 50 Ω is obtained by adjusting the gap between the microstrip feedline and virtual ground of the CPW configuration. The suggested antenna is realized using the Rogers RT/Duroid 6002 substrate, which has a loss tangent of 0.0012 and a relative permittivity of 2.94. The proposed antenna has a compact size of W_1_ × L_1_ × H = 32 mm × 25 mm × 1.52 mm. Moreover, the results were verified by using the electromagnetic (EM) software High Frequency Structural Simulator (HFSSv9). The optimized parameters of the proposed ultra-wideband antenna are given below:

W_1_ = 32, W_2_ = 11, W_3_ = 1.5, W_4_ = 5, W_5_ = 8, L_1_ = 25, L_2_ = 5, L_3_ = 8, L_4_ = 4, L_5_ = 2, L_6_ = 9.5, R = 8.5, H = 1.52; all units are in millimeters (mm).

### 2.2. Design Stages of UWB Antenna

**Figure 2:**
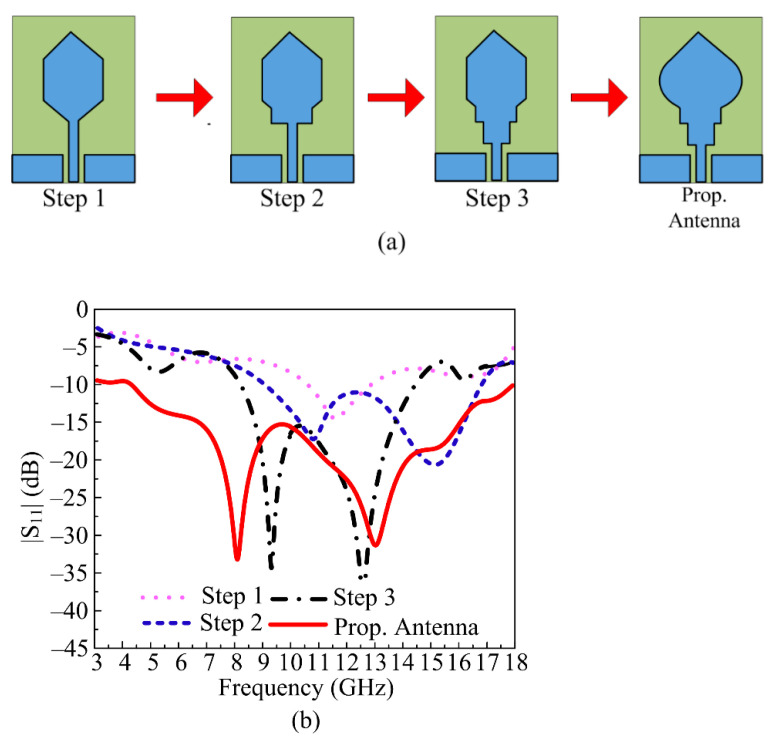
(**a**) Various design steps of proposed UWB antenna: (**b**) effect of design stages on |S_11_| parameter.

In order to obtain the required antenna characteristics, various design steps were carried out to obtain the final proposed antenna geometry operating at ultra-wideband. In the first step, the hexagonal patch antenna with CPW feedline was designed for the central frequency of 12 GHz. The antenna has operational bandwidth of 2 GHz covering 11–13 GHz. In the second step, the rectangular stub was added between the radiating patch and the feedline. The addition of this stub increases the electrical length of the antenna, which results in an improvement in return loss and bandwidth. The antenna starts operating at 11 GHz and 15 GHz with a return loss of less than 15 dB. In the third step, another rectangular stub was added below the existing stub, as shown in Figure 2a, which results in the S-parameter of the proposed antenna becoming stable and showing dual resonances at 9.5 GHz and 13 GHz. The antenna has operational bandwidth <−10 dB at 7.5–14.5 GHz with return loss <−35 dB, as shown in Figure 2b. In the final stage, two circular stubs of radius 5 mm were loaded on both sides of the central patch. As a result, the operational bandwidth of the antenna improves from 3 GHz to 8 GHz. The resultant antenna resonates at 7.5 GHz and 13 GHz with a bandwidth of 15 GHz ranging from 3–18 GHz, as depicted in Figure 2a,b.

### 2.3. Optimization Algorithm

**Figure 3:**
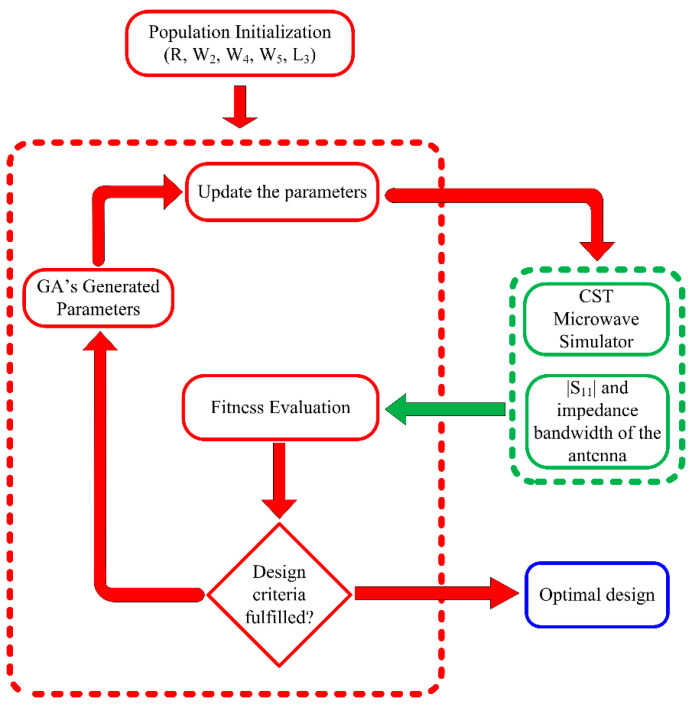
Flow chart explaining the working of genetic algorithm utilized to optimize the antenna.

A genetic algorithm (GA) was used in collaboration with the full-wave modeling tool (CST MWS) to improve the characteristics of the UWB antenna. Genetic algorithm optimizers, as is well known, are robust stochastic search techniques based on the ideas and concepts of natural selection and evolution. The optimization was completed quickly and efficiently by identifying design goals for a UWB impedance bandwidth with a low |S_11_| and identifying the antenna parameters R, W_2_, W_4_, W_5_, and L_3_. Refs. [39,40,41] have more information on the GA in antenna optimization. Figure 3 depicts the flowchart of the suggested optimization procedure.

### 2.4. Parametric Analysis of Important Parameters

**Figure 4:**
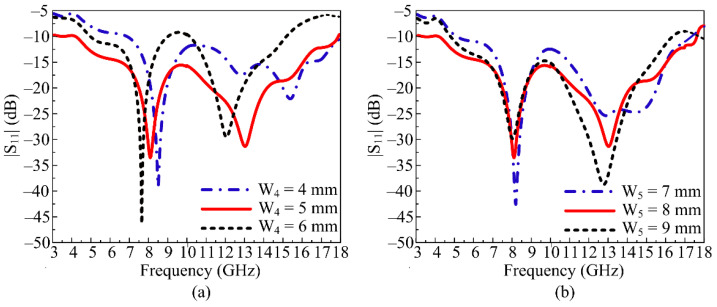
Parametric analysis of key parameters’ variation and its results: (**a**) length of the lower stub (W_4_) (**b**) length of the upper stub (W_5_).

To obtain the final geometry of the proposed UWB antenna, various design steps (as discussed above) as well as parametric analysis of important and key parameters were performed. The parametric analysis of rectangular stubs W_4_ and W_5_ is discussed in this section. The length of the lower rectangular stub (W_4_) was analyzed to observe the impact on the |S_11_| characteristic. At its optimal value of W_4_ = 5 mm, the proposed antenna offers a wideband of 5–17 GHz with resonant frequencies of 8 GHz and 13 GHz. When the value of W4 is fixed at 4 mm, the proposed antenna’s bandwidth is reduced to a dual band of 7.5–9.5 GHz and 12–17 GHz. Similarly, when the value is increased to 6 mm, again in the dual band, a slight shift towards the left side is noticed, as given in Figure 4a. The antenna offers dual frequencies ranging from 6.5–8 GHz and 11–14 GHz.

In Figure 4b, the parametric analysis of the length of the upper rectangular stub is depicted. The antenna is noticed to give a wide impedance bandwidth at the optimal value of W_5_ = 8 mm, ranging from 5–17 GHz. If the value is reduced to 7 mm, the wide bandwidth and return loss are compromised and generate dual frequency bands at 8 GHz and 14 GHz with bandwidth ranging from 7.5–9 GHz and 12.5–15.5 GHz, respectively. If W_5_ is increased to 9 mm, the suggested UWB antenna operates over 7–15 GHz, which implies a reduction of bandwidth, as shown in Figure 4b.

### 2.5. Design of Proposed Frequency Selective Surface (FSS)

**Figure 5:**
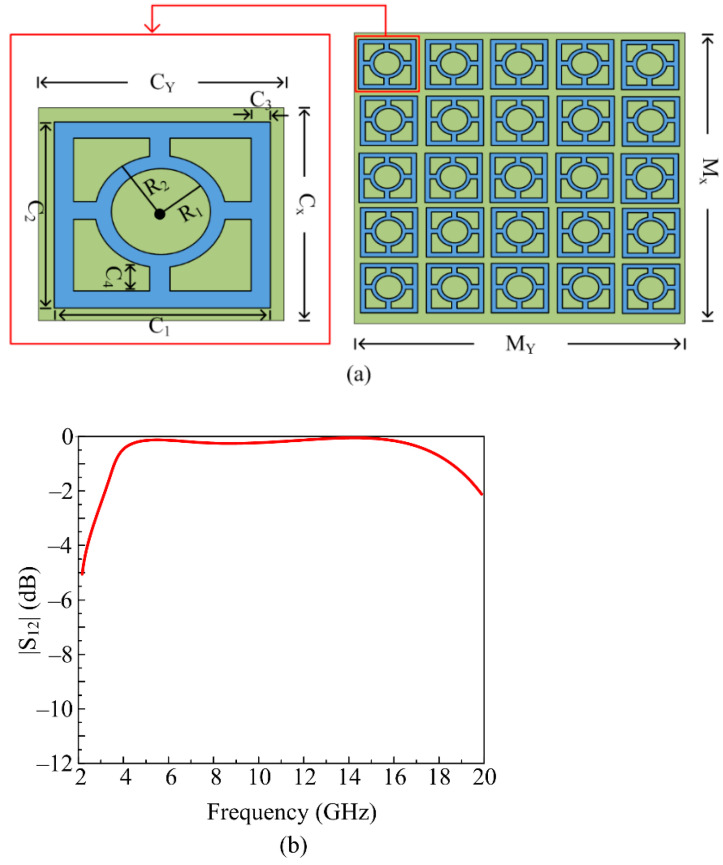
(**a**) Structure of proposed frequency selective surface (FSS) and unit cell dimensions; (**b**) |S_12_| property of suggested FSS unit cell.

To obtain the final geometry of the proposed UWB antenna, various design steps (as discussed above) were carried out. Figure 5a represents the geometrical configuration of the proposed FSS mesh as well as the unit cell. One circular ring connected with a square wall is present in the structure of each unit cell. The FSS is embedded on Rogers RT/Duroid 6002 substrate material of thickness 1.52, with relative permittivity of 2.94 and loss tangent of 0.0012. The FSS mesh contains a 5 × 5 array of 25-unit cells with a total area of *M_X_ × M_Y_* = 50 mm × 50 mm. The proposed FSS offers a wide stopband, ranging from 4–18 GHz, as given in Figure 5b. The reform parameter of the unit cell is given as: C_X_ = 10, C_Y_ = 10, C_1_ = 9, C_2_ = 9, C_3_ = 0.5, C_4_ = 1.25, R_1_ = 2, R_2_ = 2.75; all units are in millimeters (mm).

### 2.6. Proposed FSS-Loaded UWB Antenna and Its Radiation Mechanism

In this portion, the working mechanism of the proposed FSS-loaded UWB antenna is explained. The suggested antenna design is planted above the FSS sheet to reflect the radiation of the antenna coming from the back direction. The reflected wave by the FSS placed behind the antenna is in-phase with the antenna radiation, which results in an improvement in gain. The most important parameter is the distance or gap (G) between the antenna and FSS, which establishes the constructive interface of wave reflecting back from the FSS with the waves radiating from the proposed UWB antenna. The equation given below is used to adjust the gap between the antenna and FSS [42].

𝜑 − 2𝛽G = 2𝑛π, where 𝑛 = … −1, 0, 1 … (1)

**Figure 6:**
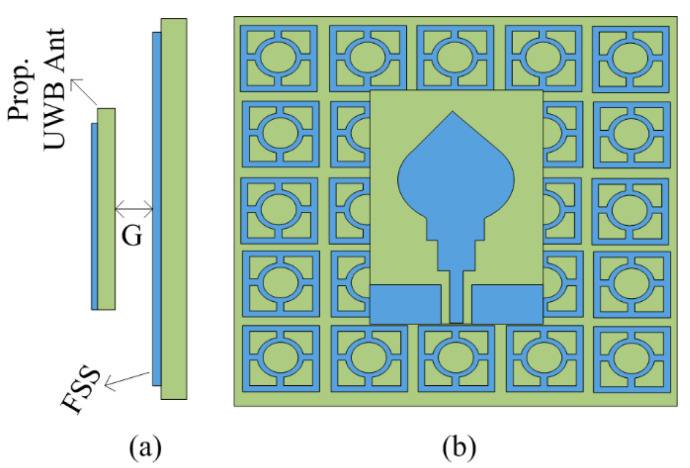
Frequency selective surface-loaded UWB antenna: (**a**) side view; (**b**) top view (G = 9 mm).

**Figure 7:**
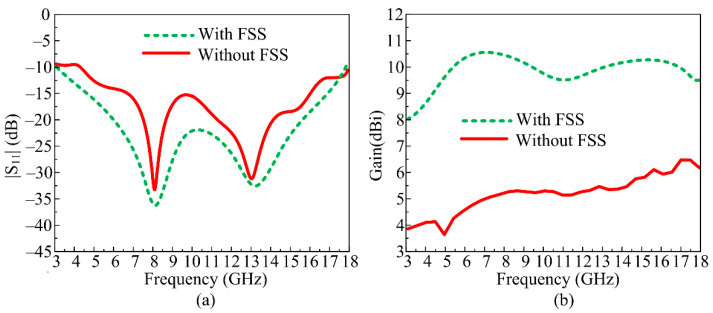
(**a**) Simulated |S_11_| plot of suggested antenna with and without FSS; (**b**) predicated gain of suggested antenna with and without FSS.

Equation (1) is composed of three parts: the reflection phase (𝜑), the free space propagation constant (𝛽), and the gap between the antenna and FSS (G), while π = 3.1415. The space in the middle of the antenna and FSS structure is optimized in order to obtain higher gain as well as wideband. In the case of the proposed work, the gap G = 9 mm. The placement of the antenna over the FSS structure is given in Figure 6a,b. The |S_11_| behavior of the suggested compact and UWB antenna in the presence and absence FSS is given in Figure 7a. It is evident that after loading, the FSS behind the antenna offers a slight improvement in impedance bandwidth. The bandwidth of the antenna improves from 12 GHz to 15 GHz, ranging from 3–18 GHz. On the other hand, the gain versus frequency plot expresses that antenna average gain improved to 19.5 dBi from 5.5 dBi after loading the FSS, as shown in Figure 7b.

## 3. Results

**Figure 8:**
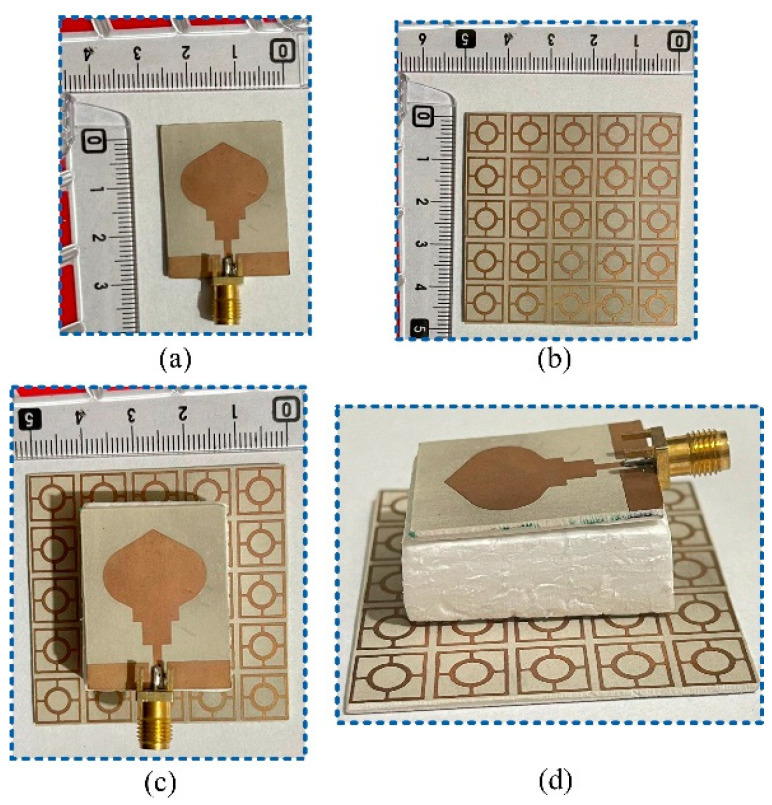
Fabricated prototype of (**a**) UWB antenna; (**b**) FSS design; (**c**) antenna placed at FSS layer; (**d**) side view of antenna placed over FSS layer.

Figure 8 depicts the hardware prototype of the proposed compact and UWB antenna as well as the FSS-loaded antenna. The S-parameters of the antenna are recorded and verified by using Vector Network Analyzer (M9375A PXI) by KEYSIGHT Tech (Santa Rosa, CA, USA), which has a 300 KHz to 26.5 GHz frequency range. Due to its negligible effect on the results of the antenna, Styrofoam of 9 mm in thickness was placed in the gap between the antenna and FSS sheet. In the shield anechoic chamber along with a horn antenna placed at a 3 m distance, the far-field results of the proposed FSS-loaded antenna were observed and verified.

### 3.1. S-Parameters

**Figure 9:**
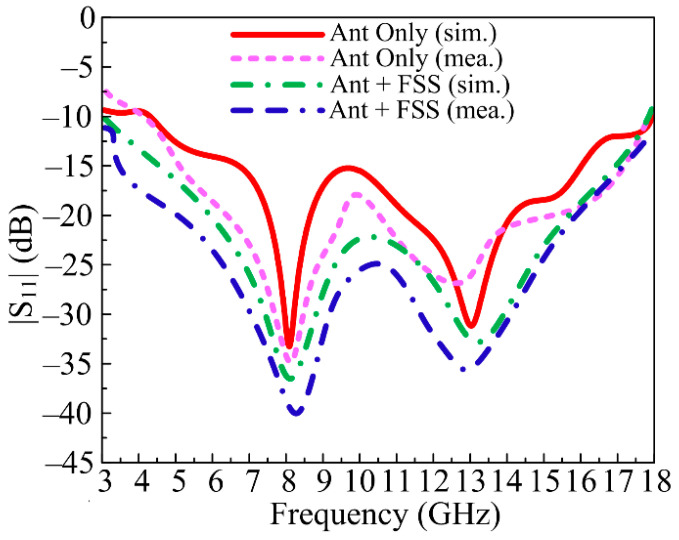
Simulated and measured S-parameter of the proposed UWB antenna with and without FSS.

In Figure 9, the contrast between the prototyped measured and software simulated scattering parameters of the suggested antenna is provided with and without FSS. The antenna offers a wideband of 12 GHz ranging from 5–17 GHz without FSS, with a resonance frequency of 8 GHz and 13.25 GHz. Meanwhile, the FSS-loaded antenna offers a wide bandwidth of 15 GHz ranging from 3–18 GHz, with resonances at 8 GHz and 13.5 GHz, as given in Figure 9. The proposed UWB antenna with and without FSS shows good agreement between measured and simulated results.

### 3.2. Gain of Antenna with and without FSS

**Figure 10:**
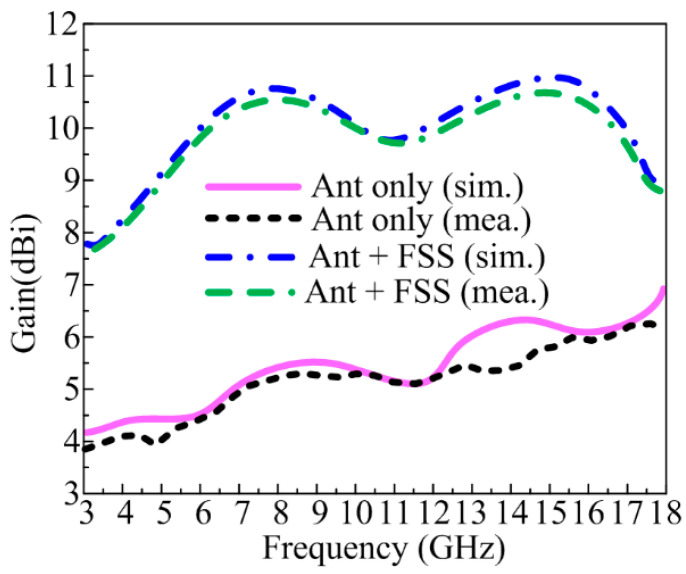
Software-predicted and prototype-measured gain of suggested UWB antenna with and without FSS.

The recommended UWB antenna’s gain versus frequency plot, either with or without FSS, is displayed in Figure 10. The suggested antenna offers a gain >5 dBi at the functional band, with a peak gain value of around 6 dBi at the resonance frequency of 13 GHz, which can be seen in Figure 10. The gain of the FSS-loaded UWB antenna was enhanced by approximately 5.5 to 6 dBi. With a peak value of 10.75 dBi and 11 dBi at resonance frequencies of 8 GHz and 13.5 GHz, respectively, the antenna with an FSS layer delivers a gain > 10 dBi at operational bandwidth, as shown in Figure 10. It is also obvious from the illustration that there are no significant disparities between the measured results and the predicted results.

### 3.3. Radiation Efficiency

**Figure 11:**
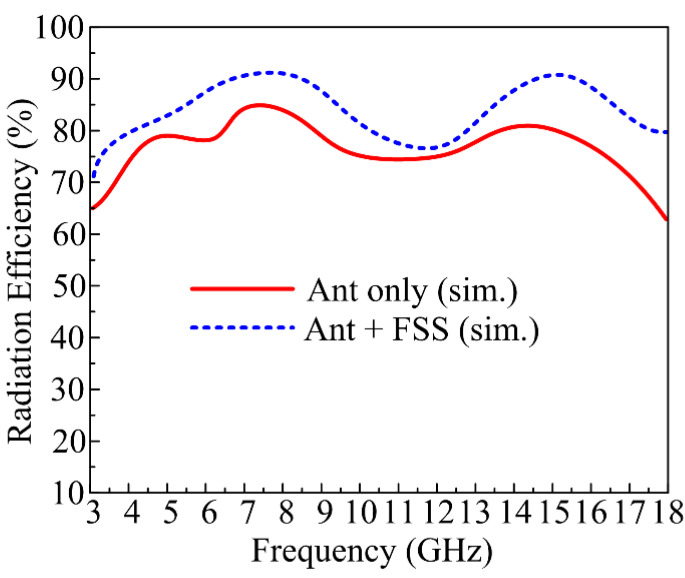
Simulated radiation efficiency of suggested UWB antenna with and without FSS.

The radiation efficiency of the suggested UWB antenna is given in Figure 11. The antenna offers radiation efficiency >75% in operational bandwidth with peak values of 83% and 82% at resonance frequencies of 7.5 GHz and 14.5 GHz, respectively. After loading the FSS layer, a slight improvement in radiation efficiency is observed. The antenna loaded with FSS offers radiation efficiency >78% at operational bandwidth with peak values of 90% at 8 GHz and 88% at 13.5 GHz.

### 3.4. Radiation Pattern of Proposed Work

**Figure 12:**
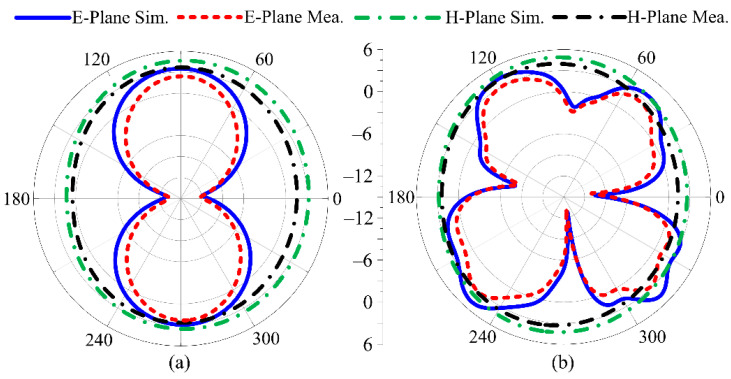
Predicted and measured radiation pattern of suggested UWB antenna without FSS at (**a**) 8 GHz; (**b**) 13 GHz.

**Figure 13:**
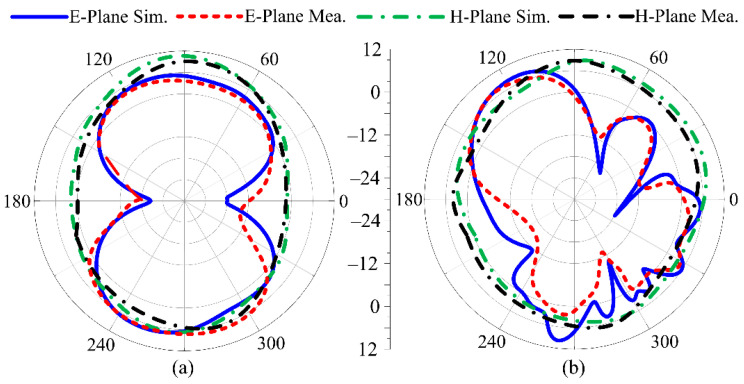
Predicted and measured radiation pattern of suggested UWB antenna with FSS at (**a**) 8 GHz; (**b**) 13 GHz.

The suggested UWB antenna’s radiation pattern at resonance frequencies of 8 GHz and 13 GHz is illustrated in Figure 12 without the application of an FSS layer. The proposed UWB antenna delivers a bidirectional radiation pattern for the E-plane at 8 GHz, but an omnidirectional radiation pattern on the H-plane at both operational frequencies. At 13 GHz, the radiation pattern is butterfly-shaped, which may be due to multiple stub insertions. The simulated results of the proposed antenna show strong agreement with the measured radiation pattern. On the other side, Figure 13 illustrates the radiation pattern of the suggested UWB antenna loaded with single-layer FSS. The radiation pattern was simulated and measured at resonance frequencies of 8 GHz and 13 GHz. The FSS at the rear side of the antenna reflects the backward radiation, due to which the broadside radiation pattern is obtained.

### 3.5. Comparison with State-of-the-Art

In Table 1, the proposed FSS-loaded UWB and the high-gain antenna are compared with antenna designs already published in the literature. When compared to other designs operating at the same frequency applications, the proposed FSS-loaded antenna is smaller in size, has a lower profile, and has a lower overall volume. The operational bandwidth and gain of the suggested FSS-loaded antenna are also higher than those of other works published in the literature. Moreover, the overall size, volume, operational bandwidth, gain, and the number of FSS layers proved that the suggested FSS-loaded antenna is a strong candidate for future 5G and 6G devices for high-gain and wideband applications.

**Table 1:** Comparison of proposed FSS-loaded antenna with work in literature operating over same frequency bands and offering gain improvement.

Ref	Overall Antenna Size (λ × λ × λ)	Volume of Antenna (mm^3^)	Operational Bandwidth (GHz)	Gain without FSS (dBi)	Gain with FSS (dBi)	No. of FSS Layers
[19]	0.31 × 0.61 × 0.1	37,210	3–11.9	3.65	7.8	Single
[20]	0.65 × 0.65 × 0.45	156,282	3.6–3.9	2	3	Single
[21]	0.5 × 0.51 × 0.1	25,190	2.9–9.3	3.12	5.12	Single
[23]	0.99 × 0.99 × 0.33	305,407	3.6–6.1	3.8	7.8	Single
[26]	0.85 × 0.85 × 0.27	195,075	3–12	2.8	6.6	Single
[27]	0.53 × 0.65 × 0.24	75,400	3.1–18.6	2.7	6.9	Single
[28]	0.75 × 0.75 × 0.15	149,737	2.5–11	6.5	8.5	Single
[30]	0.54 × 0.54 × 0.19	52,543	3.16–15	4	8.9	Single
[31]	0.44 × 0.44 × 0.2	38,720	3–14.6	4.5	8.7	Double
[32]	0.44 × 0.44 × 0.33	64,856	3.05–13.4	4.2	8.5	Double
[35]	0.48 × 0.48 × 0.3	57,600	3–21	4.8	7.2	Single
[37]	0.79 × 0.79 × 0.2	115,596	3.1–13.9	4.9	9.7	Single
[43]	0.9 × 0.9 × 0.13	53,900	3.7–11	6	9	Single
This Work	0.5 × 0.5 × 0.09	31,000	3–18	6.5	10.5	Single

## 4. Conclusions

This article presents a geometrically simple, compact, ultra-wideband (UWB) antenna with a frequency selective surface (FSS) that provides high gain. The antenna contains a simple hexagonal patch with multiple stubs inserted to obtain an ultra-wideband of 5–17 GHz. Afterwards, to reflect radiation directed backward, the FSS layer is positioned beneath the antenna to slightly improve bandwidth and enhance gain from 6.5 dB to 10.5 dB. The resultant FSS-loaded antenna offers an ultra-wideband of 15 GHz, ranging from 3–18 GHz. The FSS array contains 5 × 5 unit cells, which have an overall size of 50 mm × 50 mm. The proposed UWB antenna and FSS layer are engineered on top of Rogers RT/Duroid 6002 with a thickness of 1.52 mm. The proposed FSS-loaded UWB antenna is designed using the electromagnetic (EM) software tool High Frequency Structure Simulator (HFSS v9). The software-predicted outcomes of the suggested antenna loaded with FSS were verified with a fabricated hardware prototype. The suggested FSS-loaded UWB antenna was also contrasted with published research, demonstrating that it is a strong contender for future wireless high-gain and wideband devices.

## Data Availability

Not applicable.
